# Maternal mental health in the first year postpartum in a large Irish population cohort: the MAMMI study

**DOI:** 10.1007/s00737-022-01231-x

**Published:** 2022-04-29

**Authors:** Susan Hannon, Deirdre Gartland, Agnes Higgins, Stephanie J. Brown, Margaret Carroll, Cecily Begley, Déirdre Daly

**Affiliations:** 1 School of Nursing and Midwifery, Trinity College Dublin, 24 D’Olier Street, Dublin, DO2 T283 Ireland; 2 Intergenerational Health, Murdoch Children’s Research Institute, Melbourne (Vic), Australia; 3 Department of Pediatrics, University of Melbourne, Melbourne (Vic), Australia; 4 Department of General Practice, University of Melbourne, Melbourne (Vic), Australia

**Keywords:** Perinatal mental health, Depression, Anxiety, Stress

## Abstract

**Purpose:**

The international perinatal literature focuses on depression in the postpartum period. Prevalence and pathways of depression, anxiety and stress from pregnancy through the first postpartum year are seldom investigated.

**Methods:**

MAMMI is a prospective cohort study of 3009 first-time mothers recruited in pregnancy. Depressive, anxiety and stress symptoms measured using the Depression, Anxiety and Stress Scale (DASS 21) in pregnancy and at 3-, 6-, 9- and/or 12-months postpartum.

**Results:**

Prevalence of depressive and stress symptoms was lowest in pregnancy, increasing to 12-months postpartum. Anxiety symptoms remained relatively stable over time. In the first year after having their first baby, one in ten women reported moderate/severe anxiety symptoms (9.5%), 14.2% reported depression symptoms, and one in five stress symptoms (19.2%). Sociodemographic factors associated with increased odds of postpartum depression, anxiety and stress symptoms were younger age and being born in a non-EU country; socioeconomic factors were not living with a partner, not having postgraduate education and being unemployed during pregnancy. Retrospective reporting of poor mental health in the year prior to pregnancy and symptoms during pregnancy were strongly associated with poor postpartum mental health.

**Conclusions:**

The current findings suggest that the current model of 6-week postpartum care in Ireland is insufficient to detect and provide adequate support for women’s mental health needs, with long-term implications for women and children.

## Introduction

Although many women navigate pregnancy, birth and motherhood in good physical and mental health, it is a significant transition (Jomeen 2017; Jomeen and Martin 2008; Nelson 2013; Parfitt and Ayers 2014), and adverse mental health effects are widely recognised (Jomeen 2004; Lee 2000). Perinatal mental health problems are among the most common health issues associated with childbearing (Howard and Khalifeh 2020), and are of public health concern owing to the long-term consequences for a woman’s wellbeing (Meltzer-Brody and Stuebe 2014), intimate relationships (Yeaton-Massey and Herrero, 2019), the mother-infant bond (Cirulli et al. 2003) and the physical, social and cognitive development of her child (O’Connor et al. 2016; Schuurmans and Kurrasch, 2013). In addition, maternal suicide remains the leading cause of direct deaths occurring within a year after the end of pregnancy in the UK (Knight et al. 2021) and Ireland (O’Hare et al. 2021).

A review of longitudinal studies of antenatal and postpartum depression reports prevalence of 17.2% and 13.1% respectively (Underwood et al. 2016). Another review found higher prevalence of depression in the antenatal and postpartum periods among women in low and middle income countries (LMIC) compared to women in high-income countries (HIC) (Antenatal: LMIC = 19.2%, HIC = 9.2%; Postpartum: LMIC = 18.7%, HIC = 9.5%) (Woody et al. 2017). There is considerable variation in the depression prevalence reported between countries and cultures (Abdollahi et al. 2011; Halbreich and Karkun 2006).

While research in the perinatal period has predominately focused on depression, there is increasing interest on the impacts of anxiety, and comorbid anxiety and depression (Austin et al. 2010; Falah-Hassani et al. 2016; Farr et al. 2014). There is variation in the prevalence of anxiety disorders between the antenatal and postpartum period, between countries and cultures and between HIC and LMIC (Dennis et al. 2017) and evidence of higher prevalence of anxiety than depression both during and after pregnancy (Lee et al. 2007; Wenzel et al. 2003).

Stress is considered a distinct negative emotional state with deleterious impact on one’s overall mental health (Lovibond and Lovibond 1995a, b) and has been linked to negative outcomes for mother and child (Dunkel Schetter and Tanner 2012). Investigation of perinatal/maternal stress is frequently subsumed with anxiety and reported as ‘distress’ in the perinatal literature (Bryson et al. 2021).

A history of mental health problems is a key, and frequently cited, risk factor for poor maternal mental health outcomes (van der Waerden et al. 2015; Patton et al. 2015). Other risks include low socioeconomic status, inadequate social or emotional support, poor partner relationship, current domestic abuse, past abuse, refugee or asylum seeker status, undesirable obstetric outcomes, undesirable neonatal outcomes or an unplanned pregnancy (Fisher et al. 2012; Gartland et al. 2019; Paschetta et al. 2014).

### Perinatal mental health in Ireland

Ireland’s first National Maternity Strategy (Department of Health 2016) led to the development and publication of the Specialist Perinatal Mental Health Model of Care for Ireland (Health Service Executive (HSE) 2017). Though the model is ‘informed by national and international epidemiological evidence of need’ (HSE 2017 p7), estimates of the numbers of women affected by perinatal mental illnesses in Ireland are extrapolated from the UK data such as Prevention in Mind (Hogg 2013), and JCP-MH (2012).

Currently, in Ireland, there are no data collected or published at a national level on the prevalence, pathways, outcomes or long-term development of perinatal mental health problems. Information regarding women’s perinatal mental health comes from individual studies (Department of Health 2016). In addition, perinatal maternal health care, provided by the public sector, generally ceases for mothers at 6-week postpartum. Women who experience perinatal mental health problems beyond this period are not supported within the maternity care system, or by a system enabled to readily detect and offer treatment; rather, women must identify their own needs and seek treatment.

Researcher and clinician understanding of the current state of perinatal mental health in Ireland come from several disparate sources. Huschke et al’s (2020) recent review of the available data demonstrates substantial variation in reported prevalence. The prevalence of depression during pregnancy ranged from 1% (McAuliffe et al. 2011) to 86% (Carolan-Olah and Barry 2014), and postpartum depression from 11% (Cruise et al. 2018) to 28.6% (Cryan et al. 2001). Similarly, antenatal anxiety prevalence varied from 27.3% (Togher et al. 2017) to 75% (Carolan-Olah and Barry 2014). Importantly, the review did not identify data on postpartum anxiety prevalence. Prevalence of stress during pregnancy varied from 25% (Bennett and Kearney 2018) to 75% (Carolan-Olah and Barry 2014), with a single study reporting 8% in the postpartum period (Bennett and Kearney 2018). A possible reason for such wide variation may be differences in sample composition and size, criteria for inclusion, measurements used and assessment time points. Additionally, the authors note that only one study included a representative sample (Cruise et al. 2018).

In light of the sparse, contradictory and absent data on perinatal mental health in Ireland, there is a need for a better understanding of the prevalence, trajectories over time and risk factors for poor perinatal mental health for women giving birth in Ireland. The current study adds to the Irish and international literature using the advantages of data collect on depression, anxiety and stress at multiple time point across the first postpartum year, with a largely representative sample.

The current paper uses data from a large prospective cohort study of nulliparous women giving birth in Ireland to (1) describe the prevalence of depression, anxiety and stress during pregnancy and the first year postpartum; (2) assess changes in prevalence over the first year of motherhood and (3) identify factors associated with poor postpartum mental health.

## Methods

### Design

This study is part of the larger MAMMI (**M**aternal health **A**nd **M**aternal **M**orbidity in **I**reland) study, a multi-site, multi-strand, longitudinal cohort study examining the health of first-time mothers giving birth in Ireland. The MAMMI study is a mixed-methods study incorporating self-completion surveys, data collection from consenting women’s maternity records and one-to-one interviews with women experiencing a specific morbidity, for example sexual health problems (Daly et al. 2018; O’Malley et al. 2019, 2018) (for details on study design: https://www.tcd.ie/mammi/).

### Recruitment

Nulliparous women were recruited at their first booking visit, with the majority enrolling in the second trimester of pregnancy, (average 17.5 weeks’ gestation), from three maternity hospitals in Ireland between January 2012 and March 2017. Eligible women were nulliparous, aged 18 years and over and able to read and understand English sufficiently to complete the surveys.

Midwives in the participating recruitment sites offered all women meeting the study’s eligibility criteria a study information pack at their first antenatal appointment. Approximately 8243 women received the study information across the three research sites; 3131 participants completed the enrolment questionnaire, giving a response rate of 38%. Follow-up was at 3-, 6-, 9- and 12-months postpartum. No incentive was given for participation. Ethics approval was granted by the three hospitals and the university research ethics committees.

### Participants

**Fig. 1:**
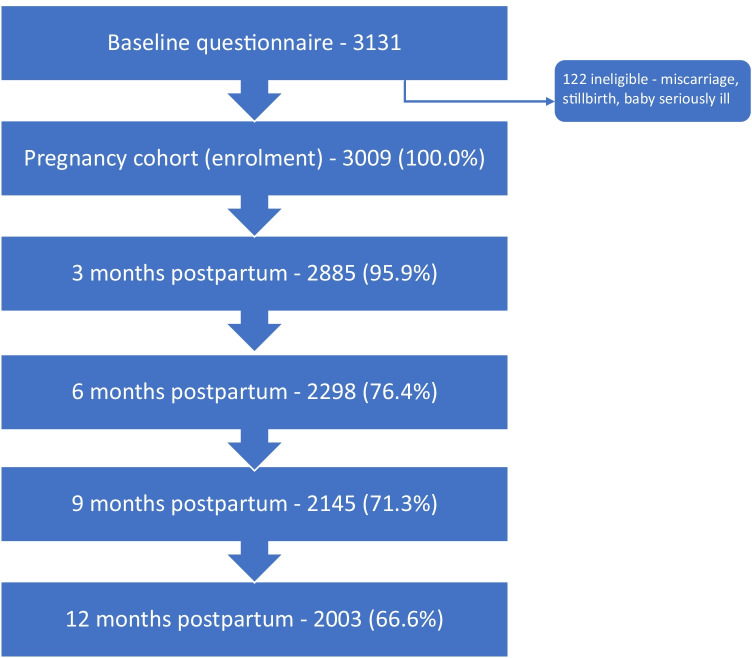
Flowchart of participation

Of the 3131 women who completed the enrolment questionnaire in pregnancy, 122 had a miscarriage, stillbirth or seriously ill baby in the NICU and were excluded. The majority of women were born in Ireland (73.8%), were aged 30 years or older (73.9%), were living with a partner (97.3%) and had postgraduate education (71.7%) (see Table 1). A flowchart of participation is presented in Fig. 1.

**Table 1:** Maternal sociodemographic characteristics and report of moderate/severe depression, anxiety or stress symptoms in the first year postpartum (*n* = 2380)

	Cohort	Depression (DASS scale ≥ 7)		Anxiety (DASS scale ≥ 6)		Stress (DASS scale ≥ 10)	
	*n* (%)	*n* (%)	OR [95% CI]^1^	*n* (%)	OR [95% CI]	*n* (%)	OR [95% CI]
Pregnancy (enrolment)							
Mothers age							
18–24 years	138 (5.8)	39 (28.5)	2.8^***^[1.8–4.2]	32 (23.5)	3.5^***^[2.2–5.4]	45 (32.6)	2.4^***^[1.7–3.6]
25–29 years	481 (20.3)	80 (16.7)	1.4^*^[1.0–1.9]	63 (13.2)	1.7^**^[1.2–2.4]	106 (22.0)	1.4^**^[1.1–1.9]
30–34 years	1081 (45.6)	136 (12.6)	1.0 [ref]	88 (8.2)	1.0 [ref]	179 (16.6)	1.0 [ref]
35 + years	673 (28.4)	78 (11.6)	0.9 [0.7–1.2]	41 (6.1)	0.7 [0.5–1.1]	122 (18.1)	1.1 [0.9–1.4]
Country of birth							
Ireland	1735 (73.8)	235 (13.6)	1.0 [ref]	152 (8.8)	1.0 [ref]	344 (19.8)	1.0 [ref]
Other EU country	470 (20)	71 (15.2)	1.1 [0.9–1.5]	45 (9.6)	1.1 [0.8–1.6]	80 (17)	0.8 [0.6–1.1]
Non-EU country	146 (6.2)	26 (17.8)	1.5 [0.9–2.1]	27 (18.5)	2.3^***^[1.5–3.7]	28 (19.2)	1.0 [0.6–1.5]
Living with partner							
Yes	2308 (97.3)	317 (13.8)	1.0 [ref]	211 (9.2)	1.0 [ref]	435 (18.8)	1.0 [ref]
No	64 (2.7)	18 (28.1)	2.4^**^[1.4–4.3]	12 (19)	2.3^*^[1.2–4.4]	19 (29.7)	1.8^*^ [1.1–3.1]
Post graduate qualification							
Yes	1695 (71.7)	209 (12.4)	1.0 [ref]	131 (7.8)	1.0 [ref]	304 (17.9)	1.0 [ref]
No	669 (28.3)	123 (18.5)	1.6^***^[1.3–2.0]	88 (13.3)	1.8^***^[1.4–2.4]	149 (22.3)	1.3^*^[1.1–1.6]
Paid employment							
Yes	2165 (91.2)	282 (13.1)	1.0 [ref]	181 (8.4)	1.0 [ref]	396 (18.3)	1.0 [ref]
No	210 (8.8)	53 (25.4)	2.3^***^[1.6–3.2]	43 (20.6)	2.8^***^[2.0–4.1]	59 (28.1)	1.7^***^[1.3–2.4]
BMI prior pregnancy							
Underweight (< 18.5)	55 (5.2)	8 (14.5)	1.1 [0.5–2.4]	6 (10.9)	1.3 [0.5–3.1]	10 (18.2)	1.0 [0.5–2.1]
Normal weight (18.5–24.9)	732 (69.1)	97 (13.3)	1.0 [ref]	64 (8.8)	1.0 [ref]	131 (17.9)	1.0 [ref]
Overweight/obese (≥ 25)	272 (25.7)	46 (17)	1.4 [0.9–2.0]	25 (9.3)	1.1 [0.7–1.7]	59 (21.7)	1.3 [0.9–1.8]
Birth outcomes (3 months postpartum)							
Weeks’ gestation at birth							
Preterm (< 36.9 weeks)	138 (5.8)	27 (19.7)	1.6^*^[1.0–2.4]	19 (14.1)	1.6 [1.0–2.7]	35 (25.4)	1.5 [1.0–2.2]
Term (37–41.9 weeks)	2191 (92.3)	298 (13.7)	1.0 [ref]	199 (9.1)	1.0 [ref]	410 (18.7)	1.0 [ref]
Post-term (≥ 42 weeks)	44 (1.9)	10 (23.3)	1.9 [0.9–3.9]	5 (11.6)	1.3 [0.5–3.4]	10 (22.7)	1.3 [0.6–2.6]
Birthweight (hospital data)							
Less than 2500 g	99 (4.4)	19 (19.4)	1.4 [0.9–2.4]	13 (13.4)	1.5 [0.8–2.7]	21 (21.2)	1.1 [0.7–1.8]
2500–3999 g	1864 (82.5)	266 (14.3)	1.0 [ref]	174 (9.4)	1.0 [ref]	370 (19.8)	1.0 [ref]
4000 g or more	296 (13.1)	36 (12.2)	0.8 [0.6–1.2]	26 (8.8)	0.9 [0.6–1.4]	50 (16.9)	0.8 [0.6–1.1]
Mode of birth (maternal report)							
Spontaneous vaginal	787 (34.3)	95 (12.1)	1.0 [ref]	75 (9.6)	1.0 [ref]	134 (17.0)	1.0 [ref]
Operative vaginal	762 (33.2)	109 (14.4)	1.2 [0.9–1.6]	54 (7.1)	0.7 [0.5–1.1]	139 (18.2)	1.1 [0.8–1.4]
Caesarean section	748 (32.6)	117 (15.7)	1.4^*^[1.0–1.8]	84 (11.3)	1.2 [0.9–1.7]	161 (21.5)	1.3^*^[1.0–1.7]
Total	2380 (100)	336 (14.2)		225 (9.5)		456 (19.2)	

^1^Odds ratio (95% confidence intervals) representing odds of reporting mental health outcome

^*^p < 0.05, ^**^p < 0.01, ^***^p < 0.001

While there is limited national data in Ireland, available data for the study recruitment period suggest that the cohort is broadly representative in terms of maternal age and country of birth (CSO 2016; Coulter-Smith 2016; IMIS 2016), but may have a higher proportion of women living with their partner, in paid work, and fewer women who had spontaneous vaginal births (see Supplementary Table 1 for more details) (Daly et al. 2019).

The sample for this paper comprised 2380 women who completed the DASS at 2 or more follow-ups in the first year postpartum. Prevalence of symptoms at each time point is presented graphically for the 1806 women who completed the DASS at all time points from pregnancy to 12-months postpartum.

### Measures

#### Self-report on mental and social factors in the year PRIOR and DURING index pregnancy

Participants were asked ‘During the last 12 months BEFORE your pregnancy did you experience…’ and ‘Since the start of your pregnancy, have you experienced…’.

- feeling depressed, low mood or sad (lasting 2 weeks or more)
- intense anxiety (such as panic attacks)
- relationship problems with your partner/spouse
- fear of a partner

Response options were ‘often’, ‘occasionally’, ‘rarely’, and ‘never’. Responses were dichotomised as often/occasionally versus rarely/never.

#### Pregnancy and birth

In the baseline questionnaire, participants were asked if the index pregnancy was conceived with assistance from fertility treatments. Response options included ‘Fertility drugs’, ‘In Vitro Fertilisation (IVF)’, ‘Intracytoplasmic sperm injection (ICSI)’ and ‘Other (please describe)’. Weeks’ gestation at enrolment and birth was calculated using the estimated due date and date of enrolment and infant birth date respectively. Mean gestation at enrolment was 17.5 weeks (range 4.0–39.7 weeks). Maternal self-report of mode of birth and infant birth weight were employed for this study, as they have been shown to be very reliable when compared to hospital records (Gartland et al. 2012).

#### Repeated mental health assessment

The short form Depression, Anxiety and Stress Scale (DASS 21) (Henry and Crawford 2005; Lovibond and Lovibond 1995a, b) was used to assess mental health symptoms at enrolment in pregnancy and at 3-, 6-, 9- and 12-months postpartum. Three scales of 7 items are rated on a 4-point scale ranging from 0 ‘Did not apply to me at all’ to 3 ‘Applied to me very much or most of the time’. The Depression scale includes dysphoria, hopelessness, devaluation of life and self-deprecation. The Anxiety scale includes autonomic arousal, skeletal muscle effects and situational anxiety. The Stress scale includes difficulty relaxing, nervous arousal and being easily upset/agitated. Good reliability and discriminant validity have been reported (Brown et al. 1997; Crawford and Henry 2003; Henry and Crawford 2005). The DASS excludes potential confounders such as sleep disturbance, appetite and weight changes, tiredness and fatigue, which may not be appropriate indicators of depression, anxiety or stress among pregnant and postpartum populations. Cronbach alpha coefficients for each subscale have been reported as very good in both pregnant (Depression; *α* = 0.82, Anxiety; *α* = 0.79, Stress; *α* = 0.89) (Xavier et al. 2016) and postpartum (Depression; *α* = 0.84, Anxiety; *α* = 0.77, Stress; *α* = 0.86) (Miller et al. 2006) populations.

At each time point, scales were summed to create a total scale score. A dichotomous variable was created for each scale to identify *none* or *low* symptoms versus *moderate* to *severe* symptoms using the recommended cut off scores: ≥ 7 for the depression scale, ≥ 6 for anxiety and ≥ 10 for stress (Lovibond and Lovibond 1995a, b). Scoring above the cut off scores indicates clinically relevant levels of psychological distress. A 12-month period prevalence variable was created for each scale to identify moderate to severe symptoms reported at one or more of the 3-, 6-, 9- or 12-month follow-ups.

### Sociodemographic and socioeconomic data

Sociodemographic information (relationship status, age, country of birth) and socioeconomic (education qualifications, employment and housing) were collected at enrolment and follow-up.

## Data analysis

Regression analyses were conducted to assess if baseline DASS scores reported by women at enrolment in pregnancy were affected by timing of enrolment in weeks’ gestation or by trimester. Prevalence of depression, anxiety and stress symptoms in pregnancy and at 3, 6, 9 and 12 months were calculated at each time point as the proportion of women reporting clinically relevant level symptoms divided by the number of women who completed the scale. Logistic regression was used to model associations between report of depression, anxiety and stress in the first year postpartum and (1) maternal sociodemographic and socioeconomic characteristics and (2) preceding social, physical and maternal factors. Models examining associations between preceding factors and postpartum mental health (2) were adjusted for sociodemographic and socioeconomic factors associated with postpartum mental health in step (1).

## Results

### Maternal mental health in pregnancy and the first year postpartum

Women completed the DASS at enrolment in pregnancy with gestations ranging from 4 to 39 weeks (mean gestation = 17.5). The majority of women enrolled in the second trimester (75.5%). Linear regression modelling identified no difference in baseline total DASS scores by weeks’ gestation (*F*_(1,2374)_ =  − 0.003, 95% CI − 0.030, 0.025, *p* = 0.854) or the odds of reporting moderate/severe depressive, anxiety or stress symptoms in pregnancy by trimester (data not shown). Therefore, DASS scores in pregnancy are presented as a single time point rather than stratified by gestation.

**Fig. 2:**
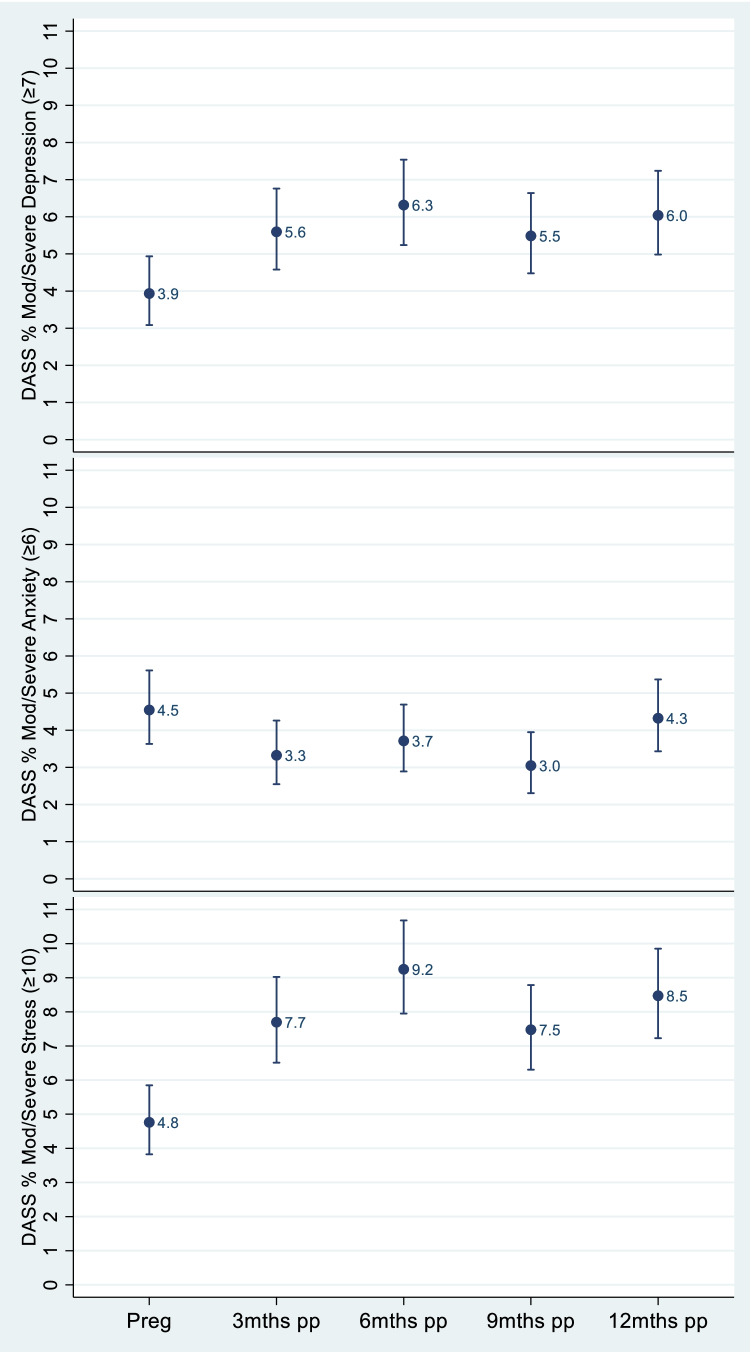
Proportion of women reporting moderate/severe depression, anxiety or stress symptoms in pregnancy and over the first year postpartum (*n* = 1806)

To examine the prevalence of moderate/severe mental health symptoms over time, a subsample of women who completed the DASS at every time point (pregnancy, and at 3-, 6-, 9-, and 12-months postpartum) are presented in Fig. 2. Different patterns in the prevalence of moderate/severe depression, anxiety and stress symptoms reported over time were observed and are described below.

As shown in Fig. 2, the proportion of women classified as having moderate/severe depressive symptoms was lowest in pregnancy (3.9%), and significantly higher at 6- and 12-months postpartum (≈6.0%). The proportion of women reporting moderate/severe anxiety symptoms was relatively consistent from pregnancy (4.5%) across the first year postpartum (≈4.0), with overlapping 95% confidence intervals indicating no statistically significant differences. Stress was different again, with the lowest proportion of women reporting moderate/severe levels of stress in pregnancy (4.8%), rising to 7.7% at 3 months postpartum and remaining significantly higher across the following time points (see Fig. 2).

### Demographic and birth factors associated with postpartum mental health

Associations between demographic factors and report of poor mental health in the first year postpartum are shown in Table 1 for the larger sample of 2380 women who completed the DASS in a minimum of 2 postpartum follow-ups.

In the first year after having their first baby, one in ten women reported moderate/severe anxiety symptoms (9.5%), more than one in ten reported moderate/severe depression symptoms (14.2%) and one in five reported moderate/severe stress (19.2%).

### Demographic factors

Being younger (< 30 years) was associated with higher odds of reporting depression, anxiety and stress symptoms in the first year postpartum. For example, women aged 18–24 years in pregnancy had higher odds of reporting depressive symptoms (OR = 2.8, 95% CI 1.8–4.2), anxiety (OR = 3.5, 95% CI, 2.2–5.4) and stress (OR = 2.4, 95% CI 1.7–3.6) compared to women in the median age (30–34 years). Being born in a non-EU country, not living with a partner, not having postgraduate education and being unemployed during pregnancy were associated with 2–3 times higher odds of reporting depressive symptoms, anxiety symptoms or stress in the first year of mothering.

### Birth factors

Having a pre-term birth was associated with higher odds of postpartum depressive symptoms, while giving birth via a caesarean section was associated with higher odds of postpartum depressive and stress symptoms. The baby’s birth weight was not associated with poor postpartum mental health.

### Health and social factors associated with postpartum mental health

#### Factors in the year prior to the pregnancy (retrospective report)

Poor mental health in the year prior to the index pregnancy was strongly associated with poor postpartum mental health as shown in Table 2. Endorsement of depressive or anxiety symptoms as happening *occasionally* or *often* in the year prior to the pregnancy was associated with a four to sevenfold increase in the odds of depressive, anxiety or stress symptoms in the first year postpartum, after adjusting for maternal age, education and relationships status. Similarly, relationship problems in the year prior to the pregnancy were associated with a twofold increase in the odds of postpartum depressive, anxiety or stress symptoms. Fertility treatment to conceive this pregnancy was not associated with poor mental health in the first year postpartum.

**Table 2:** Preceding factors and report of moderate/severe depression, anxiety or stress symptoms in the first year postpartum (*n* = 2380)

	First year postpartum (3, 6, 9 and /or 12 months)					
	Depression (DASS scale ≥ 7)		Anxiety (DASS scale ≥ 6)		Stress (DASS scale ≥ 10)	
	*n* (%)	Adj. odds ratio^1^	*n* (%)	Adj. odds ratio	*n* (%)	Adj. odds ratio
**Year prior to pregnancy (retrospective)**						
Depressed (occasionally/often vs never/rarely)	133 (43.3)	6.7^***^ [5.1–8.9]	79 (25.9)	4.3^***^[3.1–6.0]	151 (48.6)	5.2^***^ [4.0–6.8]
Anxiety (occasionally/often vs never/rarely)	79 (37.3)	3.8^***^ [2.7–5.2]	65 (30.7)	4.7^***^ [3.3–6.7]	110 (50.9)	4.9^***^ [3.6–6.5]
Relationship problems (occasionally /often vs never/rarely)	47 (28.7)	2.5^***^ [1.7–3.7]	30 (18.5)	2.2^***^ [1.4–3.5]	54 (32.7)	2.2^***^ [1.5–3.1]
SF36 item global health (good/v poor vs excellent/v good)	122 (21.4)	1.8^***^ [1.4–2.3]	87 (15.3)	1.9^***^ [1.4–2.6]	158 (27.5)	1.8^***^ [1.4–2.3]
Fertility treatment (drugs/IVF/ICSI vs no)	33 (13.9)	1.2 [0.8–1.8]	19 (8.1)	1.2 [0.7–1.9]	44 (18.4)	1.1 [0.7–1.5]
**Pregnancy (enrolment)**						
Living with a partner (no partner vs partner)	18 (28.1)	2.0^*^ [1.1–3.7]	12.0 (19.0)	1.8 [0.9–3.6]	19 (29.7)	1.6 [0.9–2.8]
Relationship problems (occasionally /often vs never/rarely)	30 (35.7)	5.6^***^ [4.0–7.8]	50 (28.1)	4.3^***^ [2.9–6.3]	81 (45)	3.9^***^ [2.8–5.4]
Fear of partner (yes vs no)	9 (40.9)	4.4^***^ [2.8–6.9]	29 (31.9)	4.5^***^ [2.8–7.4]	51 (54.8)	5.3^***^ [3.4–8.1]
Depressed (occasionally /often vs never/rarely)	79 (44.1)	2.9^***^ [1.8–4.8]	24 (28.6)	3.5^***^ [2.0–6.1]	37 (44)	3.3^***^ [2.0–5.2]
Anxiety (occasionally/often vs never/rarely)	39 (42.4)	4.2^**^ [1.7–10.2]	10 (45.5)	9.0^***^[3.7–21.9]	12 (54.5)	5.2^***^ [2.2–12.3]
DASS depression (mod to severe vs normal/mild)	71 (65.7)	12.8^***^ [8.3–19.8]	42 (38.9)	6.0^***^ [3.9–9.4]	69 (62.7)	7.6^***^ [5.0–11.5]
DASS anxiety (mod to severe vs normal/mild)	61 (45.9)	5.1^***^ [3.5–7.5]	55 (41.4)	6.9^***^[4.6–10.3]	71 (53)	5.0^***^ [3.4–7.2]
DASS stress (mod to severe vs normal/mild)	72 (56.3)	8.4^***^ [5.7–12.3]	49 (38.3)	6.0^***^[4.0–9.0]	86 (65.2)	8.7^***^ [5.9–12.8]
Total	336 (14.2)		225 (9.5)		456 (19.2)	

^1^Adjusted for maternal age, education, relationships status and recruitment site

^*^p < 0.05, ^**^p < 0.01, ^***^p < 0.001

### Factors in pregnancy

Endorsement of two items asking about depressive or anxiety symptoms during pregnancy was associated with a four to fivefold increase in the odds of depression, anxiety or stress postpartum. Moderate to severe depression, anxiety and stress symptoms (DASS) during pregnancy were associated with a five to 12-fold increase in the odds of reporting moderate to severe postpartum symptoms, with the highest odds observed for the same dimension. For example, depressive symptoms in pregnancy were associated with odds of 12.8 (95% CI 8.3–19.8) for postpartum depressive symptoms compared to women not reporting depressive symptoms in pregnancy.

Other factors during pregnancy associated with postpartum depressive, anxiety and stress symptoms included relationship problems, fear of a partner and, (for depressive symptoms only) not living with a partner.

While prior depressive or anxiety symptoms were strongly associated with postpartum mental health, it is important to note that most women with poor postpartum mental health had not reported these symptoms in pregnancy. Of women with depressive symptoms in the first year postpartum, 69.1% had not reported depressive symptoms in pregnancy (on the single item or the DASS). Similarly, 71% of women with postpartum anxiety symptoms had not reported anxiety symptoms in pregnancy.

## Discussion

In this large Irish cohort of first-time mothers, one in seven women reported moderate to severe depressive, and one in ten reported moderate to severe anxiety symptoms at some points in the first postpartum year. This study’s findings are congruent with those from other longitudinal studies of perinatal depression and anxiety reported in developed countries (Dennis et al. 2017; Underwood et al. 2016). One in five women in the MAMMI study cohort reported moderate to severe stress symptoms in their first year of mothering, congruent with a study identified from the USA, where 21% of multiparous women reported moderate/severe stress at ≈5-months postpartum (Clout and Brown 2015).

Depression, anxiety and stress were highest among younger first-time mothers (< 30 years). This is in line with international (Agnafors et al. 2019; Silverman et al. 2017) and Irish (Cruise et al. 2018) data (which was conducted with a representative sample) indicating that motherhood at a younger age represents increased risk for poor mental health. Additional factors indicating socioeconomic disadvantage were lower educational qualification attainment, not cohabiting with a partner and maternal unemployment during pregnancy and sociodemographic risk was being born in a non-EU country—these factors were all more common for the younger mothers compared to older first-time mothers. For example, 27% of mothers aged 18–24 years had postgraduate education compared to 76% of mothers aged 35 or older; similarly, 62% were working in pregnancy compared to 95% of older mothers. This study’s findings correspond with international data on socioeconomic and sociodemographic risk factors for poorer postpartum mental health (Goyal et al. 2010). It is worth noting that the findings on unemployment, lower educational qualification attainment, living apart from a partner and being born in a non-EU country highlight at risk groups who may benefit from facilitated access to perinatal mental health services and support. Socioeconomic disadvantage is linked to exposure to multiple risks with cumulative impact, such as decreased financial resources and social support, resulting in greater barriers to accessing mental health services and reduced likelihood of disclosing mental health symptoms to health care professionals (Goyal et al. 2010; Kimerling and Baumrind 2005). The sociodemographic risk implied by a woman being born outside of an EU country is of particular importance in an Irish context as the ethnic diversity of the population living and giving birth in Ireland is projected to continue to increase (CSO 2016). In addition, disparities in racial and ethnic minority access to mental health care is noted in Irish ( Bojarczuk et al. 2015) and the international literature (Cook et al. 2017).

As with reported depressive and anxiety symptoms, reported stress symptoms were more common for mothers in the younger age groups. In 2018, 62.4% of first-time mothers were aged 30 years or over, and 26% of first births were to women aged 35 years or older (HPO 2021). It may be that decreased resources available to the younger mothers are more strongly associated with stress (Easterbrooks et al. 2011), while older mothers may have more social and economic resources to buffer or protect their mental health.

Women with pre-term births had increased likelihood of experiencing symptoms of depression and stress (borderline statistical significance) in their first postpartum year. This is congruent with the literature, which reports two to six times increase in postpartum anxiety and/or depressive symptoms among mothers with pre-term infants (Farr et al. 2014).

Following adjustment for maternal age, education and relationship status, women who reported relationship problems or being afraid of their partner during the pregnancy had higher odds of experiencing moderate to severe depressive, anxiety and stress symptoms in the postpartum period. While relationship problems should not be conflated with intimate partner violence, these findings on relationship problems and fear of partner highlight that clinicians need to provide all women with an opportunistic window to discuss available supports and resource pathways, and assistance to access support where required.

The current study further adds to the international literature highlighting that mental health symptoms prior to pregnancy are strong indicators of potential postpartum issues (Dunkel Schetter and Tanner 2012; Bryson et al. 2021). Notably, in the MAMMI study cohort, a positive response to two single-item questions on the experience of depression or anxiety were associated with a four to sevenfold increase in the odds of moderate to severe postpartum depressive, anxiety or stress symptoms. Comparably, the two-item Whooley questions, which assess recent low mood and loss of interest or pleasure, have been found to have high sensitivity and modest specificity in detecting depression and high acceptability with women (Bosanquet et al. 2015; National Collaborating Centre for Mental Health 2007; Yapp et al. 2019). Integration of the current questions into routine antenatal clinical visits offers a time-efficient and potentially non-invasive means of identifying risk for both anxiety and depression symptoms among women who may benefit from closer postpartum monitoring and support. Additionally, moderate to severe symptoms during pregnancy on the DASS were associated with five to 12-fold increased odds of moderate to severe symptoms in the postpartum. The relationship between self-reported symptomatology detected by the DASS in pregnancy and subsequent increase in symptomology in the postpartum suggest that the DASS may offer a robust means of antenatal screening for a broader scope of mental health issues occurring postpartum. Suggested use of the two single-item questions or the DASS, however, are offered with the caveat that women’s disclosures in the current study were made with the expectation of anonymity rather than to a healthcare professional which may circumvent possible self-censoring that women engage in due to stigma or concerns about healthcare or child service intervention.

Although reports of depressive or anxiety symptoms during pregnancy were strongly associated with mental health problems in the postpartum, it is important to note that a majority of women who reported postpartum moderate to severe depression and anxiety symptoms did not report symptoms during pregnancy (69.1% and 71% respectively). Additionally, the current study identified variation in the prevalence of depressive, anxiety and stress symptoms across the first year, with peaks observed for depressive and stress symptoms at 6- and 12-months postpartum. The majority of women reporting postpartum symptoms without prior history and the variability in symptomatology support the need for repeated enquiry about mental health in both antenatal and postpartum care.

In Ireland, maternal postpartum care typically concludes at 6-week postpartum. This study’s findings suggest that the current model of postpartum care in Ireland is insufficient to detect, and provides adequate support for women’s mental health needs. Furthermore, women birthing in Ireland have reported that their postpartum care is predominately infant-centered and infrequently facilitates opportunities for women to discuss their own physical or mental health (Daly et al. 2021). Continued postpartum contact is vital to ensure that women who develop symptoms beyond 6-week postpartum are identified and receive the support that they need to regain optimum health.

## Strengths and limitations

The strengths of this study include the use of a robust data collection instrument, a large sample size and frequent data collection across the first postpartum year. The findings need to be considered in light of the following limitations: although the sample is broadly representative of women giving birth in Ireland in terms of nationality and age, the sample was over representative of women living with their partner and in paid work. Additionally, limited data on socioeconomic and obstetric outcomes at a national level preclude a detailed comparison of the representativeness of the cohort. Thus, the findings may not be generalisable to women beyond the predominant sample demographics.

## Conclusion

International and Irish research on perinatal mental health has focused on depression and the early postpartum months. There is limited data, particularly within an Irish context, to inform policy and practice on a broader range of mental health challenges and their development as women progress through motherhood. The current study offers findings from a large prospective Irish cohort study describing the prevalence and trajectories of depressive, anxiety and stress symptoms from pregnancy and the first year postpartum, and factors associated with poor maternal mental health. These findings are important to building a robust foundation of data on perinatal mental health in Ireland to inform national action.

This study’s findings suggest there are a substantial proportion of first-time mothers who experience depressive, anxiety and stress symptoms in pregnancy and the first postpartum year. Indices of sociodemographic and socioeconomic disadvantage indicate that younger mothers, women without higher education, women who are unemployed and women not living with a partner in pregnancy are at increased risk for reporting poor postpartum mental health, as are women who report relationship problems or fear of a partner.

The variation in mental health symptoms across the first year postpartum and new onset of symptoms imply that women would benefit from prolonged contact and support from maternity care services beyond the 6-week postpartum that is currently the norm for maternal care in Ireland.

Given the well-documented adverse impact of poor postpartum mental health for a woman’s wellbeing, her functioning as a mother and the cognitive and emotional development of her child (George et al. 2013; Cirulli et al. 2003; Yeaton-Massey and Herrero 2019; O’Connor et al. 2016; Schuurmans and Kurrasch 2013; Surkan, Kennedy, Hurley, Black, 2011; Hay, Pawlby, Waters, Perra, Sharp 2010), clinicians and researchers should be mindful that maternal mental health encompasses more than the experience of depression. Clinicians need also to be attentive to identifying and meeting women’s mental health needs through facilitation of disclosure, appropriate knowledge of (Higgins et al. 2018) and referral to, care and treatment pathways to ensure timely care of women.
